# Low self-esteem and the formation of global self-performance estimates in emerging adulthood

**DOI:** 10.1038/s41398-022-02031-8

**Published:** 2022-07-11

**Authors:** Marion Rouault, Geert-Jan Will, Stephen M. Fleming, Raymond J. Dolan

**Affiliations:** 1 Institut Jean Nicod, Département d’études cognitives, ENS, EHESS, CNRS, PSL University, 75005 Paris, France; 2 Laboratoire de neurosciences cognitives et computationnelles, Département d’études cognitives, ENS, INSERM, PSL University, 75005 Paris, France; 3 Department of Clinical Psychology, Utrecht University, Utrecht, The Netherlands; 4 Wellcome Centre for Human Neuroimaging, University College London, London, UK; 5 Max Planck UCL Centre for Computational Psychiatry and Ageing Research, University College London, London, UK; 6 Department of Experimental Psychology, University College London, 26 Bedford Way, London, WC1H 0AP UK

**Keywords:** Human behaviour, Depression

## Abstract

High self-esteem, an overall positive evaluation of self-worth, is a cornerstone of mental health. Previously we showed that people with low self-esteem differentially construct beliefs about momentary self-worth derived from social feedback. However, it remains unknown whether these anomalies extend to constructing beliefs about self-performance in a non-social context, in the absence of external feedback. Here, we examined this question using a novel behavioral paradigm probing subjects’ self-performance estimates with or without external feedback. We analyzed data from young adults (*N* = 57) who were selected from a larger community sample (*N* = 2402) on the basis of occupying the bottom or top 10% of a reported self-esteem distribution. Participants performed a series of short blocks involving two perceptual decision-making tasks with varying degrees of difficulty, with or without feedback. At the end of each block, they had to decide on which task they thought they performed best, and gave subjective task ratings, providing two measures of self-performance estimates. We found no robust evidence of differences in objective performance between high and low self-esteem participants. Nevertheless, low self-esteem participants consistently underestimated their performance as expressed in lower subjective task ratings relative to high self-esteem participants. These results provide an initial window onto how cognitive processes underpinning the construction of self-performance estimates across different contexts map on to global dispositions relevant to mental health such as self-esteem.

## Introduction

A positive view of the self is a crucial determinant of mental health [1, 2]. Low self-esteem has been associated with a number of psychiatric conditions, particularly those of an anxious and depressive nature [1, 2]. People form beliefs about themselves and their abilities (“self-beliefs”) across many levels of abstraction, ranging from local confidence in individual decisions to estimates of performance on an entire task, up to global estimates about their own worth as expressed in reports of self-esteem. Having positive beliefs about self-worth (i.e., high self-esteem) is associated with a stronger ability to successfully deal with prospective situations, including how one deals with day-to-day challenges [3]. For instance, people with low self-esteem are often faster to disengage from a task in response to failure than those with high self-esteem [4]. Despite the recognized importance of self-beliefs for mental health, surprisingly little is known about the precise cognitive building blocks of self-beliefs, and their relationship with self-esteem [5].

Recent work examining the construction of momentary self-worth from social feedback [6, 7] has started to uncover the formation of self-beliefs in a social context. Here low self-esteem participants were slower to update beliefs about how much others liked them, and faster to update momentary feelings of self-worth in response to social feedback. These findings provide initial evidence of a differential construction of self-beliefs being tied to a more global, stable construct such as self-esteem [6, 7]. However, it remains unclear whether this is a specific idiosyncrasy of how low self-esteem individuals construct self-worth from social feedback or, alternatively, whether low self-esteem individuals have a domain-general bias when forming appropriate self-beliefs that extend to other non-social contexts. One possibility is that individuals with low self-esteem may maintain a negative self-view by consistently underestimating their abilities despite performing as well as those with high self-esteem, indicating a disconnection between a “local” self-evaluation on a given task and a “global” self-evaluation such as self-esteem.

Here we examined the formation of subjective self-performance estimates in participants with high and low self-esteem, in contexts with and without explicit feedback about performance. We leveraged a recently developed behavioral paradigm probing the formation of subjective self-performance estimates [8, 9]. The main finding from this previous work is that decision confidence is a key factor contributing to the formation of self-performance estimates in the absence of feedback, a situation that echoes many real-life settings. We observed that decision difficulty, fluctuations in decision accuracy, and whether participants received feedback about their decisions all impacted their self-performance estimates. The present study employed this protocol to ask whether such subjective self-performance estimates, formed over the scale of minutes, relate to self-esteem. We previously proposed a hierarchical framework of metacognitive evaluation in which self-esteem may act as a global prior for generating self-performance estimates on a given task [10]. Specifically, under such a hierarchical framework of metacognitive evaluation – spanning decision confidence formed at a local level to self-esteem at a global level—we would expect self-esteem to provide a global context or prior for how self-performance estimates are formed on a given task [5]. We assume that self-esteem is a global estimate formed across longer timescales of months or years, whereas self-performance estimates are formed more rapidly, over the course of a few minutes of engaging in a task. Characterizing how these two constructs intersect is important to identify neurocognitive building blocks underpinning constructs relevant to mental health, such as self-esteem, and in turn facilitate novel interventions for disorders that are linked to altered self-esteem, a canonical example being depression [11, 12].

To address these questions, we capitalized on a large dataset from a well-characterized community sample of adolescents and emerging adults (*N* = 2402; aged 14 to 24 at first measurement) who reported on their self-esteem across 1–3 timepoints spanning 4.5 years. We selected low and high self-esteem participants (aged 18–25) from the larger sample as individuals who scored within the bottom, or top, 10% of a self-esteem distribution so as to maximize power for detecting individual differences due to self-esteem [7]. A comparison between high and low self-esteem individuals was motivated by well-established findings that individuals with high self-esteem rates are among the healthiest in terms of low levels of depression and high levels of well-being in the population [13], providing a strong contrast against those with low self-esteem who experience substantial problems. Participants performed short blocks of two interleaved perceptual tasks and at the end of each block, they then selected the task on which they considered they had performed best and provided a subjective ability rating about each task. These two measures enable a window onto subjective self-performance estimates [8].

Consistent with previous findings, we found that participants underestimated their own performance in the absence of feedback, despite performing equivalently in situations with and without feedback. Participants with low self-esteem rated their performance lower compared to those with high self-esteem, despite task performance being similar in the two groups. We discuss the findings within a framework in which local metacognitive variables, such as decision confidence, influence the construction and maintenance of global self-esteem across longer times-scales.

## Materials and methods

### Participants

We tested 62 human participants from the Neuroscience in Psychiatry Network (NSPN) cohort (*N* = 2402) who reported on their mental health, including measures of self-esteem, across 4.5 years for 1–3 measurements [7]. The NSPN 2400 Cohort is a general population sample of adolescents and emerging adults (*N* = 2402; aged 14–24 years at baseline) originally established to investigate a developmental change in mental health, cognition, and the brain (see ref. [14] for an in-depth cohort profile). Participants from Cambridgeshire and Greater London reported on sociodemographic characteristics and a range of mental health indices across multiple timepoints. A subsample (*N* = 785) nested within the larger cohort participated in detailed behavioral assessments of cognition using computerized tasks, clinical assessments, and IQ tests (see e.g., ref. [15]). A subset of this latter sample (*N* = 318) additionally underwent measures of brain structure and function using MRI (see e.g., ref. [16]).

For recruitment based on self-esteem, we used scores on the Rosenberg self-esteem scale (RSES) [17]. Mean RSES score of the large sample was 19.7 (on a scale of 0–30; SD = 5.62). We invited 184 participants with average RSES scores within the bottom decile (0–12) and top decile (27–30) of the large sample for further study and tested 53 participants (29 with low self-esteem; 24 with high self-esteem). To reach our target sample size of 30 participants in each group, we invited a further 51 participants whose recent RSES score was within the bottom or top decile of RSES scores and tested an additional ten participants. Five low self-esteem participants reported being in remission from a mental health problem for at least 3 years at the moment of testing. Participants were originally recruited for an fMRI study reported in (Will et al., 2020). The sample size was set to surpass the sample sizes of prior fMRI studies examining inter-individual differences in self-esteem (ten studies; median *N* = 26; range = 17–48 [18–27]). We further increased our power to detect individual differences by employing a targeted recruitment approach focusing on the extremes of a reported self-esteem distribution. After taking a break following MRI scanning, participants completed the self-performance estimate task reported here.

We matched groups based on gender and age, but not for subclinical symptoms of depression and anxiety [7, 14]. As expected from the known associations between self-esteem and depression [2], we found strong correlations between the Rosenberg self-esteem score and the MFQ depression score (ρ(55) = −0.86, *p* = 1.77 × 10^−17^), and between the Rosenberg self-esteem score and the Trait Anxiety score (ρ(55) = −0.86, *p* = 2.03 × 10^−17^). When comparing the two groups to the large cohort (*N* = 2402), we observed that the low self-esteem group is at the 78.5 (±33.3) percentile in terms of depressive symptoms, while the high self-esteem group is at the 16.4 (± 6.1) percentile. In terms of well-being the low self-esteem group is at the 16.4 ± (0.04) percentile, while the high self-esteem group is at the 85 ± (0.04) percentile. These observations suggest that to characterize self-esteem-related problems, it is informative to contrast those who manifest such problems (i.e., low self-esteem participants) with those who have few such problems (i.e., high self-esteem participants).

Other inclusion criteria were applied: no current neurological or psychiatric disease, an address in London, no color-blindness and no contraindications to MRI (as the participants also underwent MRI scanning [7]). Five participants were excluded for responding at chance level (two participants, both high self-esteem), always selecting the same rating (one participant, low self-esteem), or failing the comprehension test of the rating scale during the practice (two participants, both high self-esteem), leaving *N* = 57 participants for data analysis. The final sample consisted of 29 low self-esteem participants (mean age = 21.2, SD = 2.2; 18 women) and 28 high self-esteem participants (mean age = 21.1, SD = 2.3; 14 women). Participants were paid 8 GBP per hour for their participation and compensated for travel expenses. They provided written informed consent according to procedures approved by the London – Westminster NHS Research Ethics Committee (15/LO/1361).

### Experimental design

#### Learning blocks

**Fig. 1:**
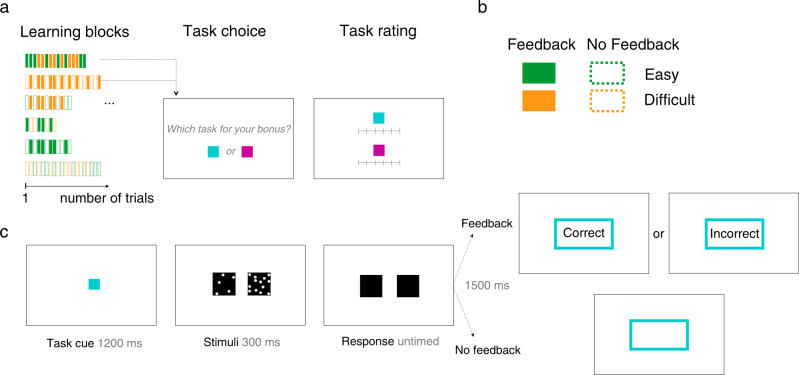
Experimental design probing the construction of self-performance estimates adapted from ref. [8]. **a** Participants performed short learning blocks of randomly alternating trials from two tasks (between 2 and 10 trials per task). At the end of each block, participants were asked to select the task on which they thought they had performed best (Task choice), as well as rate their overall ability at each task (Task rating). A new block ensued with two new color cues indicating two new tasks. **b** Each task required perceptual choices as to which of two boxes contained more dots. Trials were either easy or difficult according to the numerical dot difference between the left and right boxes. Following their response participants either received veridical feedback (correct, incorrect) about their perceptual judgment, or no-feedback. These four conditions resulted in six possible task pairings as displayed in a. **c** Each trial consisted in a perceptual judgment as to which of two boxes contained a higher number of dots, followed or not by provision of feedback.

Participants performed short learning blocks that randomly interleaved two “tasks” identified by two arbitrary color cues (Fig. 1a). Participants were incentivized to learn about their own performance on each of the two tasks over the course of a block. Each block contained 2, 4, 6, 8, or 10 trials per task (which we refer to as “*learning duration*”), giving 30 blocks (=360 trials) per participant, presented in a pseudo-random order. We varied the learning duration to examine whether and how the number of decisions made by participants within each block impacted the construction of self-performance estimates.

Each task required a perceptual judgment as to which of the two boxes contained more dots (Fig. 1a). The difficulty level of the judgment was controlled by the difference in dot number between boxes. Any given task (as indicated by the color cue) was either easy or difficult and provided either veridical feedback or no-feedback (Fig. 1b). Importantly the color cues allowed participants to identify the two tasks but provided no information about task difficulty. These four task features provided six possible pairings of tasks in learning blocks. The order of blocks was randomized for each participant.

#### Two measures of self-performance estimates

1. Task choice. At the end of each block, participants were asked to choose the task for which they thought they performed best (Fig. 1a). Specifically, they were asked to report which task they would like to perform in a short subsequent “test block” in order to gain a bonus. This procedure aimed to reveal self-performance estimates, because participants should choose the task they expect to be more successful at in the test block in order to gain maximum reward. To indicate their task choice, participants responded with two response keys that differed from those assigned to perceptual decisions to avoid any carry-over effects. The subsequent test block contained six trials from the chosen task (not shown in Fig. 1). No-feedback was provided during test blocks.
2. Task ratings. After the test block, participants were asked to rate their overall performance on each of the two tasks on a rating scale ranging from 50% (“chance level”) to 100% (“perfect”) to obtain explicit, parametric reports of self-performance estimates (Fig. 1a). Ratings were made with the mouse cursor and could be given anywhere on the continuous scale. Intermediate ticks for percentages 60, 70, 80, and 90% correct were indicated on the scale but without verbal labels. There was no time limit on perceptual choices, task choices, and task ratings. After each block, participants were offered a break and could resume at any time, with the next learning block featuring two new tasks cued by two new colors. The present design is a modified version of the protocol from our original paper [8].

*Trial structure*. Each block featured two tasks, with each trial starting with a color cue presented for 1200 ms, indicating which of the two tasks will be performed in the current trial (Fig. 1). The stimuli were black boxes filled with white dots randomly positioned and presented for 300 ms, during which time participants were unable to respond. We used two difficulty levels characterized by a constant dot difference, but the spatial configuration of the dots inside a box varied randomly from trial-to-trial. One box was always half-filled (313 dots out of 625 positions), whereas the other contained 313 + 24 dots (difficult conditions) or 313 + 60 dots (easy conditions). The position of the box with the most dots was randomized across trials (half of the trials on the left, half of the trials on the right). Participants were asked to judge which box (left or right) contained more dots and the chosen box was then highlighted for 300 ms. Afterward, a colored rectangle (cueing the color of the current task) was presented for 1500 ms. The rectangle was either empty (on no-feedback trials) or contained the word “Correct” or “Incorrect” (on feedback trials), followed by an ITI of 600 ms.

### Statistical analyses

To examine the influence of our experimental factors on self-performance estimates, we carried out three 2 × 2 × 2 repeated-measures ANOVAs on (1) objective performance (Table S1), (2) task choice (Table S2), and (3) task ratings (Table S3). Our factors were Feedback (present vs. absent), Difficulty (easy vs. difficult) as within-subject factors, and self-esteem level (high vs. low) as a between-subject factor. Because task choice frequencies are proportions, they were transformed using a classic arcsine square-root transformation before being entered into the ANOVA. Note that we reproduced these analyses based on past self-esteem status (as per recruitment) instead of current self-esteem status (on the testing session) and found virtually identical results (Tables S4–S6).

Since objective performance naturally fluctuates even for a fixed difficulty level due to noise, we examined whether participants had some insight into these fluctuations. We additionally examined whether participants’ self-performance estimates reflected fluctuations in objective performance on a given learning block over and above variations in difficulty level. For each of the six pairings, we analyzed task choice and task ratings as a function of the absolute difference in performance between tasks for each participant (as in [8]) (Fig. 3). To quantify these effects, we performed a logistic (resp. linear) regression to further quantify the influence of fluctuations in objective performance on task choice (resp. task ratings), entering objective performance as block-wise regressors. We further introduced individual self-esteem (Rosenberg score) and its interaction with the difference in performance as additional regressors to examine if these could explain additional variance in task choice or task ratings. Regressors were z-scored to ensure comparability of regression coefficients. Each model was specified as *Task Choice ~ β*_*0*_ + *β*_*1*_ × Difference in Performance + *β*_*2*_ × Self-esteem + *β*_*3*_ × Difference in Performance × Self-esteem, and participants were treated as a fixed effect in the regressions (due to few blocks per pairing per participant).

Finally, to visualize whether there were any effects of learning duration (the number of trials per task in each block) on self-performance estimates, task choice frequencies were averaged across participants for each of the six possible pairings and the five possible learning durations (Fig. 4). To investigate whether learning duration had a significant influence on task choice, separate logistic regressions were performed on each of the six task pairings. Each model was specified as *Task Choice ~ β*_*0*_ + *β*_*1*_ × Learning Duration + *β*_*2*_ × Self-esteem + *β*_*3*_ × Learning Duration × Self-esteem (we continue to model the main effects of self-esteem on each individual task pairing but do not further test for the significance of these terms, as this effect is evaluated in the more powerful ANOVA approach above that collapses over task pairings). Similarly, we examined whether learning duration influenced task ratings with similar models as for task choices, but with linear regression models instead of logistic regressions, because the dependent variable was continuous rather than dichotomous. Our dependent variable was the difference in task ratings between the two tasks of a block. The use of a fixed-effects approach naturally limits the extent to which our findings can be generalized to the population level.

## Results

### An experimental protocol probing the formation of self-performance estimates

To investigate the impact of self-esteem on self-performance estimates, participants (*N* = 57) engaged in 30 short learning blocks (4 to 20 trials) of two randomly interleaved visual discrimination tasks signaled by two arbitrary color cues (Fig. 1c). We varied learning duration (the number of trials per task in each block) to examine whether participants differentially formed self-performance estimates depending on how much experience they had with each task. Each task required a perceptual discrimination judgment as to which of the two boxes contained a higher number of dots (Fig. 1c). Two factors controlled task characteristics: task difficulty (either easy or difficult according to dot difference between boxes), and receipt of either veridical feedback (correct, incorrect) or no-feedback about performance on each perceptual choice (Fig. 1b). This factorial design resulted in six possible task pairings for learning blocks (Fig. 1a). For example, an Easy-Feedback task could be paired with a Difficult-Feedback task, or a Difficult-Feedback task could be paired with a Difficult-No-Feedback task, and so forth. At the end of each block, participants selected the task on which they believed they performed better (Task choice) and were rewarded on the basis of their performance on the chosen task (see Methods). They additionally provided a subjective rating of self-performance on each of the two tasks on a continuous scale (Task ratings) (Fig. 1a). A short break ensued before the next learning block started when two new color cues indicated two new tasks. The two end-of-block measures, namely task choices and task ratings, provided proxies for self-performance estimates. In this way the design allowed us to compare self-performance estimates in participants with high or low self-esteem.

### Self-esteem and self-performance estimates

We first examined whether high and low self-esteem participants differed in terms of objective performance on the perceptual tasks, conditional on the provision of feedback or not, and on task difficulty. We performed a 2 × 2 × 2 repeated-measures ANOVA on objective performance with two within-subject factors (Feedback and Difficulty), and with the self-esteem group as a between-subject factor (see Methods). First, we replicated our previous findings showing that participants performed better when tasks were easier (the main effect of Difficulty, *F*(1, 56) = 472.7, *p* = 1.1 × 10^−28^), but without a difference in performance in the presence or absence of feedback (Fig. S1) (main effect of Feedback, *F*(1, 56) = 0.622, *p* = 0.434). High (*N* = 28) and low (*N* = 29) self-esteem participants did not differ in performance (main effect of Self-Esteem, *F*(1, 56) = 1.675, *p* = 0.201). We found no significant interactions, except for interaction between Difficulty and Self-Esteem (*F*(1,56) = 5.174, *p* = 0.027), driven by slightly worse performance on easy tasks in the low self-esteem group (Table S1). Pairwise comparisons between each of the four experimental conditions showed no significant difference in performance in the easy conditions (*t*_*55*_ = 1.82, *p* = 0.07 for feedback trials and *t*_*55*_ = 1.55, *p* = 0.12 for no-feedback trials). There was also no statistically significant difference between groups in the difficult condition with feedback (*t*_*55*_ = 0.75, *p* = 0.45) nor in the difficult condition without feedback (*t*_*55*_ = −0.04, *p* = 0.96). Together with a lack of the main effect of self-esteem on performance, these results suggest that any difference in self-performance estimates between self-esteem groups is likely to arise at a metacognitive level, rather than being driven by systematic differences in objective performance between groups across all experimental conditions of the design.

Next, we examined the construction of self-performance estimates in our perceptual tasks. We again applied the same 2 × 2 × 2 repeated-measures ANOVA, this time to task choices and task ratings. For our first measure of self-performance estimates, task choice, we replicated our prior work showing participants selected easy tasks as compared to difficult tasks more often at the end of blocks (main effect of Difficulty *F*(1, 56) = 108.8, *p* = 1.2 × 10^−14^). Participants were also more likely to select tasks that provided feedback, compared to those that did not (main effect of Feedback *F*(1, 56) = 93.8, *p* = 1.7 × 10^−13^). There was a trend-level interaction between Difficulty and Feedback (*F*(1, 56) = 3.81, *p* = 0.056), in accordance with previous findings showing an interaction in a subset of previous datasets (Fig. S1) [8]. This we assume reflects variability in how sensitive participants are to difficulty relative to feedback receipt. We found no main effect of Self-Esteem on task choice (*F*(1, 56) = 0.295, *p* = 0.59) and no significant interactions between Self-Esteem and other experimental factors (all *p* > 0.33), meaning that task choices were most likely driven by experimentally manipulated factors as opposed to (task-unrelated) self-esteem. We also note that task choices can be insensitive to overall shifts in self-performance estimates across both tasks, which can cancel out when participants have to choose between pairs of tasks. This might be the case in low compared to high self-esteem individuals, for instance. To test for such effects, we next turned to our second measure of self-performance estimates, task ratings.

**Fig. 2:**
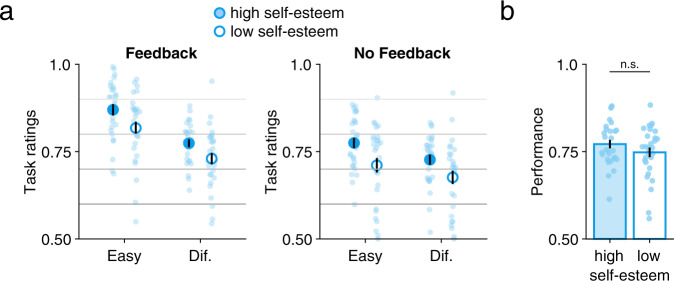
Effects of self-esteem on task ratings and performance. **a** A 2 × 2 × 2 repeated-measures ANOVA revealed a main effect of self-esteem status on task ratings, indicating lower self-performance estimates in participants with low self-esteem. Difficulty (Easy, Diff) and Feedback (Feedback, No–Feedback) were within-subject factors and self-esteem was a between-subject factor (see Methods and Results). Circles and error bars represent mean and SEM across participants (*N* = 28 with high self-esteem and *N* = 29 with low self-esteem), and dots indicate individual data points. **b** Average objective performance across all conditions for high and low self-esteem groups separately. Bars and error bars indicate mean and SEM across participants and dots indicate individual data points. n.s. not significant (two-sample *t*-tes*t*, *t*_*55*_ = 1.29, *p* = 0.20).

Finally, we analyzed our second measure of self-performance estimates: subjective task ability ratings. Consistent with previous findings [8], we found a main effect of Difficulty (*F*(1, 56) = 211.7, *p* = 1.7 × 10^−20^) and of Feedback (*F*(1, 56) = 139.9, *p* = 9.7 × 10^−17^) on task ratings, together with a significant interaction between these factors (*F*(1, 56) = 35.6, *p* = 1.8 × 10^−7^). These results indicate that participants rated their self-performance lower in the absence of feedback, an effect exacerbated for easy as compared to difficult tasks (Fig. 2 and S1). Crucially we observed a main effect of Self-Esteem on task ratings (*F*(1, 56) = 5.92, *p* = 0.018), reflecting the fact that participants with low self-esteem reported lower self-performance estimates for both difficult and easy tasks as well as tasks with and without feedback, despite their objective task performance being equivalent to participants with high self-esteem (Table S3).

Study participants were recruited on the basis of their self-esteem score at the time of inclusion in the database (“past” self-esteem level). We also assessed their self-esteem level at the time they performed the perceptual learning tasks (“current” self-esteem level) in order to create the groupings for the analyses reported above. Past self-esteem scores at the time of recruitment correlated with current self-esteem scores at the time of testing (ρ(55) = 0.72, *p* = 2.6 × 10^−12^). Nevertheless, to examine the robustness of our findings, we reproduced all our analyses but now based on past self-esteem groupings instead of current self-esteem groupings. Critically we found virtually identical results (Tables S4–S6), indicating that our behavioral task interacts with self-esteem status in a stable manner. In particular, participants with lower self-esteem at the time of recruitment continued to provide lower subjective task ratings at the time of testing, despite objective performance being unaffected (Table S6).

### Characterization of the influence of task factors on self-performance estimates

**Fig. 3:**
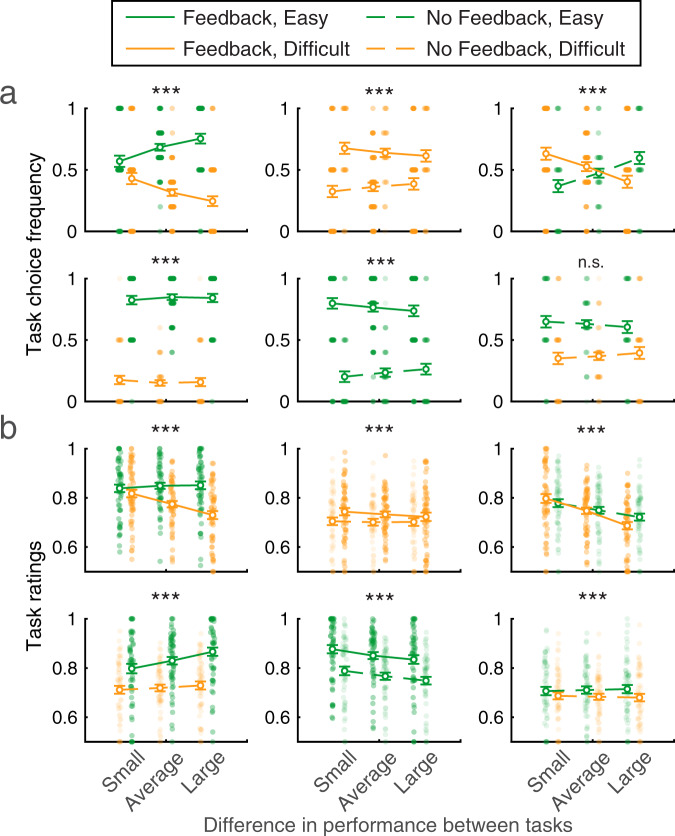
Participants’ self-performance estimates were sensitive to difficulty level, objective performance, and feedback presence. Self-performance estimates were measured as **a** task choice and **b** task ability ratings, each visualized here as a function of the absolute difference in performance between tasks, for a small, average, and large absolute difference in performance between tasks. Green (resp. orange) indicates easy (resp. difficult) tasks. Dotted lines (resp. full lines) indicate tasks without feedback (resp. with feedback). Error bars indicate SEM across participants (*N* = 57). Dots indicate individual data points; note that for task choices, task choice frequency took discrete values due to a limited number of data points per participant (see Methods). Significant effects of the difference in performance between tasks were found for end-of-block task choices (***p < .000001), except for when an easy-no-feedback task was paired with a difficult-no-feedback task (n.s.). For task ratings, there was a significant effect of the difference in performance between tasks in all blocks (***p < 0.0023).

**Fig. 4:**
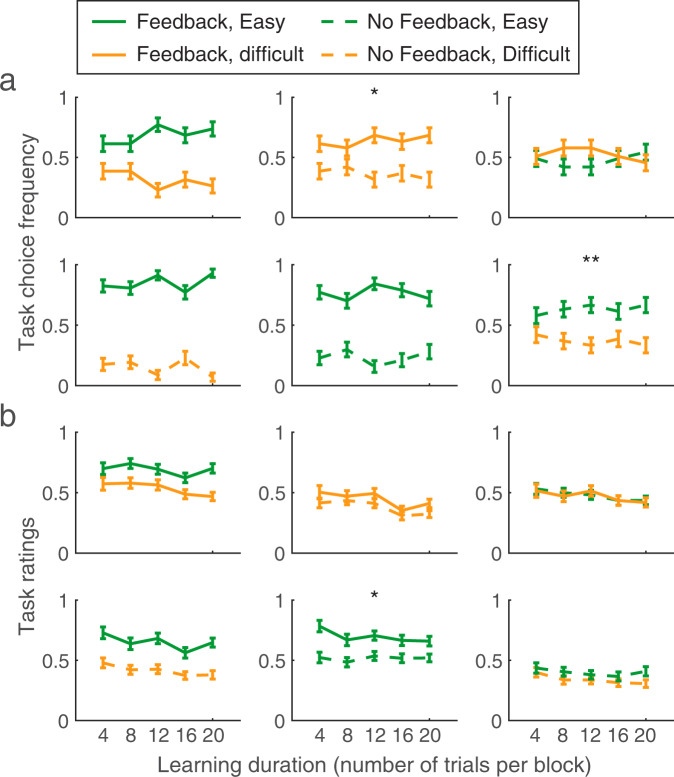
Participants’ self-performance estimates as a function of learning duration. Self-performance estimates measured as end-of-block task choices (**a**) or task ratings (**b**) as a function of learning duration (number of trials per task in each block) for the six possible task pairings. Error bars indicate SEM across participants (*N* = 57). Significant effects of learning duration on end-of-block self-performance estimates are indicated (*p < 0.05, **p < 0.01) (see Methods).

Having shown that self-esteem is linked to overall self-performance estimates in our task, we next characterized how participants’ self-performance estimates are influenced by block-to-block fluctuations in learning duration and performance and asked how the influence of these factors may interact with self-esteem. Building on our previous study [8], our experimental design with variable block lengths allowed us to characterize how experimental factors explain variation in subjective task ratings and examine if other previous findings replicate. In a first analysis, we reasoned that, even for a fixed difficulty level, there would be fluctuations in objective performance from block to block due to variability inherent to perceptual decision-making. To investigate whether participants were sensitive to such fluctuations when they provided self-performance estimates, we performed regression analyses predicting task choices and task ratings from the difference in objective performance between tasks (Fig. 3; see Methods). In a second analysis, we examined the influence of learning duration on the expression of self-performance estimates (Fig. 4; see Methods). In both these sets of analyses, we included self-esteem as an additional between-participant predictor and asked how it interacted with (i) the difference in objective performance between tasks and (ii) learning duration.

First, in the total sample (*N* = 57), we found a significant effect of a difference in performance between tasks on end-of-block task choices (all task pairings *β* = 1.09, all *p* = 1.56 × 10^−7^), except for when an Easy-No-Feedback task was paired with a Difficult-No-Feedback task (*β* = 0.083, *p* = 0.49) (Fig. 3a). These differences in performance did not interact with self-esteem, demonstrating that performance fluctuations continue to influence self-performance estimates irrespective of self-esteem level (interaction between self-esteem and difference in performance; all task pairings *β* < 0.25, all *p* > 0.21).

Using a similar approach, we uncovered a significant effect of differences in performance between tasks on end-of-block task ratings (Fig. 3b) (all task pairings *β* > 0.016, *p* < 0.0023), meaning that the larger the difference in objective performance between tasks, the larger the difference in task ratings (irrespective of self-esteem). When examining interactions with self-esteem, we found that effects of fluctuations in performance did not differ as a function of self-esteem level for the majority (five out of six) of task pairings (interaction between self-esteem and difference in performance; all *β* *<* −0.0067, *p* > 0.38). An exception was when an Easy-No-Feedback task was paired with a Difficult-Feedback task, for which the interaction between self-esteem and performance difference was significant (*β* = 0.016, *p* = 0.022), without an effect of self-esteem itself (*β* = −0.011, *p* = 0.088). This interaction indicates that participants with high self-esteem showed a greater influence of performance difference for this task pairing. Taken together, these findings indicate that participants’ end-of-block self-performance estimates were sensitive to fluctuations in objective difficulty, the presence of feedback, and fluctuations in task performance, with limited effects of self-esteem on these relationships.

Second, we examined the impact of learning duration (the number of decisions per task in each block) on self-performance estimates. Consistent with previous findings [8], regression analyses confirmed no significant effect of learning duration (number of trials per block) on end-of-block task choices for four out of six of the task pairings (all *β* < 0.21, all *p* > 0.099) (Fig. 4a). An exception was when a Difficult-Feedback task was paired with a Difficult-No-Feedback task, with learning duration leading task choices to become less sensitive over time (*β* = −1.49, *p* = 0.013). Similarly, when an Easy-No-Feedback task was paired with a Difficult-No-Feedback task, we found a significant effect of learning duration (*β* = −1.6, *p* = 0.009) which interacted with self-esteem (*β* = −1.04, *p* = 0.026) on task choices. For all other five out of six task pairings, there were no significant interactions with self-esteem (all *β* < 0.12, all *p* > 0.34).

Finally, we found no effect of learning duration on end-of-block task ratings for five out of six of the task pairings (Fig. 4b) (all *β* < 0.012, all *p* > 0.16), with the exception of when an Easy-Feedback task was paired with an Easy-No-Feedback task (*β* = −0.018, *p* = 0.033). We also found no interactions between self-esteem and learning duration (abs(*β*) < 0.012, all *p* > 0.15) on task ratings. Together these findings indicate that participants’ self-performance estimates were mostly insensitive to task duration, suggesting participants rapidly form an estimate of their expectations of success at the beginning of each block of trials and that the manner in which they do so is relatively insensitive to self-esteem level.

## Discussion

Humans construct beliefs about themselves and their abilities across many levels of abstraction, encompassing not only global constructs such as self-esteem but also self-performance estimates on a given task [5]. Having a favorable appraisal of oneself is a key component of mental well-being [1, 2]. We previously proposed a hierarchical framework in which self-esteem acts as a global prior to self-performance estimates for a given task [10]. Here, we sought behavioral evidence that bears on this framework by relating self-esteem, a global construct, to subjective self-performance estimates created during tasks performed over a shorter temporal duration. To do this we leveraged a perceptual task for which we previously characterized how participants provide self-performance estimates [8].

We replicated our previous findings showing participants’ self-performance estimates are sensitive to task difficulty, feedback, and fluctuations in objective task performance. We further showed that participants with low self-esteem provide lower subjective task ratings than those with high self-esteem, in the absence of a main effect of self-esteem on objective performance. We compared a low self-esteem group with substantial problems to a healthy group of high self-esteem participants. Low self-esteem subjects’ propensity to consistently rate their performance as worse relative to those with high self-esteem—despite not performing any worse on objective measures—represents a candidate correlates of poor mental health. This disconnect between objective performance and its subjective evaluation may therefore be relevant for a better understanding of psychiatric disorders characterized by distortions in self-evaluation, as we further discuss below.

An important feature of our results is the absence of systematically lower performance in participants with low self-esteem across all experimental conditions of the design. This indicates a selective and consistent link between self-esteem and biases in confidence, uncontaminated by differences in performance. However, we did find a small, but significant, interaction between Difficulty and Self-Esteem in first-order task performance. One possible explanation for this effect is that lower expectations of self-performance may lead participants to engage less effort in the task, and thus display worse perceptual performance. In turn, such a cycle could become reinforcing, with lower perceptual performance further decreasing subjective task ratings. However, given the absence of systematic differences in performance between each of the four conditions of the design, we consider this alternative hypothesis less likely in light of our entire set of findings. More generally, this decoupling indicates that differences in global self-performance estimate stemmed from a metacognitive bias as opposed to a rational updating of confidence as a function of objectively lowered performance. This is a key insight as while previous reports have indicated that low self-esteem individuals also underestimate their performance on familiar tasks, it has remained unclear whether this is a consequence of negative self-beliefs, or due to negative experiences with the task at hand (for a review, see ref. [28]).

In the present study, a lack of a clear influence of low self-esteem on performance may reflect participants’ having limited experience with the perceptual task. We leveraged the fact that presumably, nobody had encountered the current perceptual task before in order to minimize prior beliefs about expected performance, thereby allowing us to isolate a ‘pure’ effect of self-esteem. Instead, had it been a memory task, for instance, participants might have retrieved and relied on general prior beliefs about their memory abilities [29]. The type of perceptual task we exploit is also likely to preclude influences seen in other cognitive domains, such as mathematics anxiety [30] or pervasive social effects such as stereotype threat (a perceived risk of confirming negative stereotypes about abilities associated with one’s social group) that are thought to influence subsequent performance [31, 32]. Therefore, it is possible that relationships between self-esteem and task self-performance estimates may become even tighter in real-life metacognitive evaluations.

A key advantage of the current metacognitive task is that this difference can be interpreted through the lens of differential contributions to self-performance estimate formation. Although we cannot draw strong conclusions from non-significant findings, the lack of systematic statistical interactions between self-esteem and experimental factors (feedback presence, difficulty level) on self-performance estimates indicates participants with low self-esteem were not impaired in building self-performance estimates from task-specific factors. Specifically, they were also able to update self-performance estimates in the absence of feedback, indicating that they preserve an ability to track fluctuations in local confidence. Instead, participants with low self-esteem displayed a general underestimation of their performance as seen in subjective task ratings, independently of feedback condition and difficulty level. This result is consistent with a recent study showing that overall confidence in a perceptual task was associated with self-esteem score, in the absence of a relationship between self-esteem and metacognitive sensitivity (i.e., how well confidence tracks performance in the absence of feedback) [33]. Other studies have reported that self-esteem affects sensitivity to feedback, suggesting that high self-esteem may act as a ‘buffer’ against negative feedback [34]. Low self-esteem participants were found to provide self-worth ratings that are more sensitive to social evaluative feedback [7] or achievement feedback [34], although the type of feedback and task scope were substantially different from those of the current study. More generally, our results show that low and high self-esteem individuals continue to form global confidence estimates in a similar manner despite continuing to differ in their overall evaluation. This result is non-trivial and helps to delineate the source of confidence biases in self-esteem (as a generalized bias that appears to go beyond the influence of local task factors).

Many clinical and subclinical psychiatric symptoms are associated with alterations to various aspects of metacognition [35, 36]. Low self-esteem is a robust predictor of concurrent and future mental health disorders, particularly those associated with negative cognitions and affect as expressed in anxious and depressive symptoms [1]. Importantly, the participants in our sample did not have a formal mental health diagnosis, providing some evidence that the observed associations between self-esteem and lower subjective performance ratings are likely to be explained by low self-esteem alone, rather than factors associated with patient status, such as stigma, the impact of therapy or medication. Notably, a previous study reported that self-esteem predicted overall confidence on a perceptual task in an online general population sample, even after controlling for depressive symptoms [33]. As is typically the case, here and in our previous study [7], self-esteem groups differed on trait anxiety, state anxiety, depression, and social anxiety, reflecting existing associations between low self-esteem and these symptoms. Indeed, in the DSM-V, a lowered sense of self-worth is one of the core diagnostic criteria for major depressive disorder. Likewise, self-esteem and self-efficacy are typically strongly decreased in depression and anxiety disorders. The ecological validity of our sample, therefore, does not allow us to distinguish a specific impact of self-esteem from unique shifts in co-morbid anxiety or depression levels, which are known to be tightly linked in longitudinal studies [2]. In another study using a dimensional approach, we identified lower levels of trial-by-trial decision confidence in subclinical participants who displayed higher scores on an “anxious-depression” transdiagnostic dimension [37]. It remains to be explored whether this alteration in decision confidence might generalize to more global aspects of metacognition, such as the self-performance estimates measured here [38]. However, to the extent that self-esteem is related to negative affective symptoms, the present results showing a link between low self-esteem and lower subjective task ratings provides initial evidence this may indeed be the case. Furthermore, in previous work, we have shown that global SPEs in a similar task are sensitive to trial-to-trial fluctuations in decision confidence [8, 9]—suggesting factors that influence baseline decision confidence are also likely to influence global metacognition.

Similarly, previous work has provided evidence that other aspects of metacognition are shifted in the context of negative affective symptoms. A previous study of social anxiety documented a lack of a positivity bias—a tendency to overweight positive as compared to negative feedback—when processing feedback from a social task involving giving a speech in front of judges [39]. To the extent that social anxiety and low self-esteem are linked, these results suggest a similar lack of a positivity bias in learning may be linked to low self-esteem. Finally, in a face discrimination task participants with high anxiety manifest different feedback-related negativity correlates in EEG recordings following evaluative feedback, as compared to participants with low anxiety [40]. This indicates that anxiety might disrupt an evaluative component of performance monitoring, which we expect would extend to low self-esteem to the extent that anxiety and lowered self-esteem overlap. Another previous study provided empirical evidence that participants with depression differed in their cognitive reappraisal of positive information, suggested to be underpinned by a reduced integration of positive prediction errors [41]. In light of unexpected positive feedback about their own performance on a test, healthy participants positively updated their beliefs, whereas participants with depression did not change these task expectations [42]. To the extent that low self-esteem and depression overlap, a similar mechanism could partly explain our findings: participants with low self-esteem may not update their self-performance estimates following positive feedback as much as those with high self-esteem. However, we note that the impact of task factors on the formation of global estimates did not differ between self-esteem groups, so this hypothesis remains to be tested.

Replicating ours and others’ previous studies we found that task choices were sensitive to fluctuations in performance [8, 9, 43], an effect that remained when controlling for self-esteem level. We note that, unlike task ratings, the need to make binary task choices forces participants to separate between higher and lower self-performance estimates, even if self-performance on both tasks are close at the end of a block. This implies that any baseline shift in self-performance estimates that is common to both tasks may not manifest in task choices—possibly explaining why only ratings, but not choices, were sensitive to self-esteem level. We also replicate our previous result that learning duration did not systematically affect task choices [8] and extend this finding to the case of task ratings (Fig. 4). Although here we did not measure participants’ precision or confidence in their subjective task ratings, it is possible that uncertainty around expected performance decreases with learning duration, as participants have more samples to inform their self-performance estimates. Our analyses compared participants with high and low self-esteem levels. It would be interesting to examine whether there are any non-linear relationships between task factors, self-performance estimates, and self-esteem in a sample consisting of low, average, and high self-esteem participants.

While here we investigated the formation of global self-performance estimates over the course of short learning blocks, future work is needed, particularly using longitudinal measurements, to examine how global self-performance estimates develop over longer timescales [2] and impact subsequent metacognitive judgments [44]. This can provide a window onto the formation and maintenance of global dispositions that evolve across weeks or months, such as self-esteem itself [5]. In the present study, participants spanned a limited age range and it remains unknown how the formation of experimental self-performance estimates mirrors the maintenance and update of self-esteem across the lifespan. Some of these effects may be specific to adolescence, as previous work has shown that perceptual metacognitive sensitivity continues to mature in the 11-17 years old range [45]. In our sample, only a few people (<10%) shifted their reported self-esteem sufficiently to move between the self-esteem groups over the course of a couple of years. Nevertheless, it remains to be established how malleable such self-constructs are, though the lack of interactions between self-esteem and feedback in our experiment suggests a certain degree of stability or rigidity. Under a hierarchical framework, it is plausible that higher, more global, levels are more temporally stable whereas lower levels such as local decision confidence or self-performance estimates on individual tasks may be more malleable [5, 46].

Metacognition operates across many levels of abstraction, from local confidence in individual decisions to self-performance estimates on a particular task, to global self-evaluations such as self-esteem. However, the relationships among these levels remain to be characterized. Our approach was to recruit participants from a community sample and use a task for which participants had no prior experience, academic stakes, or relevance, enabling us to isolate an effect of self-esteem on self-performance estimates that was distinct from other factors typically present in patient studies. Our study, therefore, connects two of these levels of metacognition in a simple lab-based task, disconnected from real-life evaluations, and finds that low self-esteem is associated with lower subjective performance estimates.

## Supplementary information

Supplementary Material

## Data Availability

Participants’ group-level data for statistical analyses are available at https://www.github.com/marionrouault/RouaultWillFlemingDolan/. Participants did not provide written consent regarding the posting of their anonymized data on public repositories; however, the raw datasets are available from the corresponding author upon reasonable request.

## Code availability

MATLAB code for statistical analyses are available at https://www.github.com/marionrouault/RouaultWillFlemingDolan/.
