# Supplementary material for: Low self-esteem and the formation of global self-performance estimates in emerging adulthood
Source: Transl Psychiatry. 2022 Jul 11;12:272. doi: 10.1038/s41398-022-02031-8 (PMC9276660; doi:10.1038/s41398-022-02031-8)
Supplement: Supplementary file 1 — Supplementary Material [file 41398_2022_2031_MOESM1_ESM.pdf]

Supplementary Material

Low self-esteem and the formation of global self-performance estimates in emerging adulthood

Marion Rouault[1,2,*], Geert-Jan Will[3,*], Stephen M. Fleming[4,5,6] and Raymond J. Dolan[4,5]

[1]*Institut Jean Nicod, Département d'études cognitives, ENS, EHESS, CNRS, PSL University, 75005 Paris, France.*

[2]*Laboratoire de neurosciences cognitives et computationnelles, Département d'études cognitives, ENS, INSERM, PSL University, 75005 Paris, France.*

[3]*Department of Clinical Psychology, Utrecht University, Utrecht, The Netherlands.*

[4]*Wellcome Centre for Human Neuroimaging, University College London, London, UK.*

[5]*Max Planck UCL Centre for Computational Psychiatry and Ageing Research, University College London, London, UK.*

[6]*Department of Experimental Psychology, University College London, 26 Bedford Way, London WC1H 0AP, UK.*

[*]Equal contribution

Correspondence: marion.rouault@gmail.com

Supplementary Figures

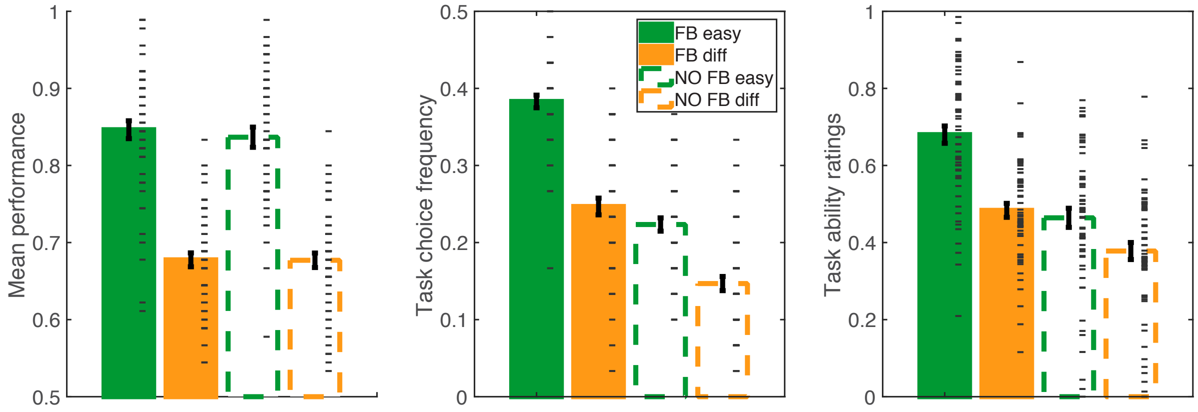

Figure S1. Behavioral dissociation between objective performance (left panel) and self-performance estimates, measured as end-of-block task choices (middle panel) and task ratings (right panel).). Green (resp. orange) indicates easy (resp. difficult) tasks. Dotted lines (resp. full bars) indicate tasks with no feedback (resp. with feedback). Error bars indicate S.E.M. across participants (N=57) with black ticks indicating individual data points. See also Results. Note that task ratings are between 0 and 1 due to non-parametric z-score within participant.

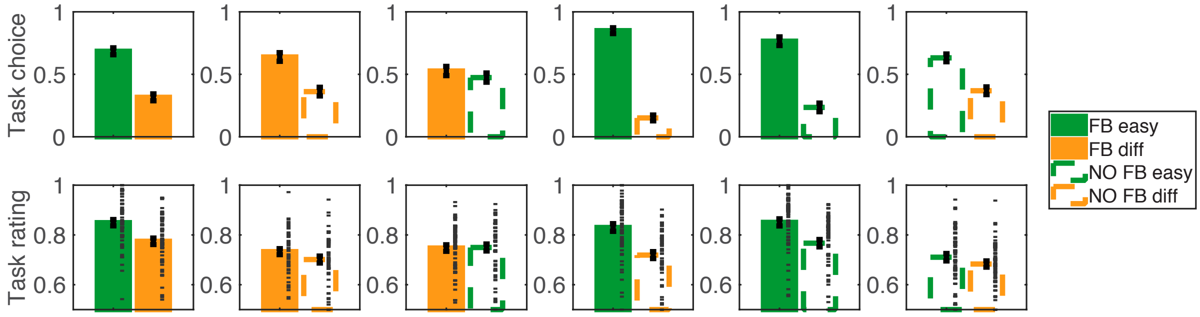

Figure S2. Self-performance estimates assessed at the end of blocks via task choices (upper panels) and task ratings (lower panels). Green (resp. orange) indicates easy (resp. difficult) tasks. Dotted lines (resp. full bars) indicate tasks with no feedback (resp. with feedback). Error bars indicate S.E.M. across participants (N=57) with black ticks indicating individual data points.

Supplementary Tables

Within Subjects Effects

	Sum of Squares	df	Mean Square	F	p
Difficulty	1.543	1	1.543	472.719	< .001
Difficulty × Self-Esteem	0.017	1	0.017	5.174	0.027
Residual	0.179	55	0.003		
Feedback	0.002	1	0.002	0.622	0.434
Feedback × Self-Esteem	8.636×10^{-4}	1	8.636×10^{-4}	0.344	0.560
Residual	0.138	55	0.003		
Difficulty × Feedback	0.001	1	0.001	0.630	0.431
Difficulty × Feedback × Self-Esteem	6.326×10^{-4}	1	6.326×10^{-4}	0.342	0.561
Residual	0.102	55	0.002		

Note. Type III Sum of Squares

Between Subjects Effects

	Sum of Squares	df	Mean Square	F	p
Self-Esteem	0.032	1	0.032	1.675	0.201
Residual	1.045	55	0.019		

Note. Type III Sum of Squares

Table S1: A 2 × 2 × 2 repeated measures ANOVA on performance with Difficulty (Easy, Difficult) and Feedback (Feedback, No Feedback) as within-subject factors and Self-Esteem (high, low) as a between-subject factor revealed a main effect of task difficulty on performance, as expected. No other main effects or interactions were significant (see Results).

Within Subjects Effects

	Sum of Squares	df	Mean Square	F	p
Difficulty	3.819	1	3.819	108.779	< .001
Difficulty × Self-Esteem	0.035	1	0.035	0.987	0.325
Residual	1.931	55	0.035		
Feedback	5.431	1	5.431	93.775	< .001
Feedback × Self-Esteem	0.004	1	0.004	0.073	0.788
Residual	3.185	55	0.058		
Difficulty × Feedback	0.128	1	0.128	3.808	0.056
Difficulty × Feedback × Self-Esteem	0.004	1	0.004	0.130	0.720
Residual	1.846	55	0.034		

Note. Type III Sum of Squares

Between Subjects Effects

	Sum of Squares	df	Mean Square	F	p
Self-Esteem	2.968×10^{-4}	1	2.968×10^{-4}	0.295	0.589
Residual	0.055	55	0.001		

Note. Type III Sum of Squares

Table S2: A 2 × 2 × 2 repeated measures ANOVA on end-of-block task choices with Difficulty (Easy, Difficult) and Feedback (Feedback, No Feedback) as within-subject factors and Self-Esteem (high, low) as a between-subject factor revealed a main effect of Difficulty and Feedback factors on task choice. No other main effects or interactions were significant (see Results).

Within Subjects Effects

	Sum of Squares	df	Mean Square	F	p
Difficulty	2521.945	1	2521.945	211.732	< .001
Difficulty × Self-Esteem	16.407	1	16.407	1.377	0.246
Residual	655.106	55	11.911		
Feedback	3259.154	1	3259.154	139.926	< .001
Feedback × Self-Esteem	11.768	1	11.768	0.505	0.480
Residual	1281.056	55	23.292		
Difficulty × Feedback	361.370	1	361.370	35.629	< .001
Difficulty × Feedback × Self-Esteem	0.880	1	0.880	0.087	0.769
Residual	557.838	55	10.143		

Note. Type III Sum of Squares

Between Subjects Effects

	Sum of Squares	df	Mean Square	F	p
Self-Esteem	1581.984	1	1581.984	5.915	0.018
Residual	14709.837	55	267.452		

Note. Type III Sum of Squares

Table S3: A 2 × 2 × 2 repeated measures ANOVA on end-of-block task ratings with Difficulty (Easy, Difficult) and Feedback (Feedback, No Feedback) as within-subject factors and Self-Esteem (high, low) as a between-subject factor revealed a main effect of Difficulty and Feedback factors on task ratings, with a significant interaction between these factors, together with a main effect of Self-Esteem. No other main effects or interactions were significant (see Results).

Within Subjects Effects

	Sum of Squares	df	Mean Square	F	p
Difficulty	1.543	1	1.543	486.237	< .001
Difficulty × Self-Esteem	0.022	1	0.022	6.864	0.011
Residual	0.175	55	0.003		
Feedback	0.002	1	0.002	0.627	0.432
Feedback × Self-Esteem	0.001	1	0.001	0.497	0.484
Residual	0.138	55	0.003		
Difficulty × Feedback	0.001	1	0.001	0.654	0.422
Difficulty × Feedback × Self-Esteem	2.580×10^{-4}	1	2.580×10^{-4}	0.139	0.711
Residual	0.102	55	0.002		

Note. Type III Sum of Squares

Between Subjects Effects

	Sum of Squares	df	Mean Square	F	p
Self-Esteem	0.014	1	0.014	0.740	0.394
Residual	1.062	55	0.019		

Note. Type III Sum of Squares

Table S4: A 2 × 2 × 2 repeated measures ANOVA on performance with Difficulty (Easy, Difficult) and Feedback (Feedback, No Feedback) as within-subject factors and Self-Esteem (high, low) as a between-subject factor. This is the same analysis as reported in Table S1 but with recruitment self-esteem level instead of current self-esteem level.

Within Subjects Effects

	Sum of Squares	df	Mean Square	F	p
Difficulty	3.819	1	3.819	108.691	< .001
Difficulty × Self-Esteem	0.033	1	0.033	0.946	0.335
Residual	1.932	55	0.035		
Feedback	5.420	1	5.420	94.699	< .001
Feedback × Self-Esteem	0.042	1	0.042	0.733	0.396
Residual	3.148	55	0.057		
Difficulty × Feedback	0.130	1	0.130	4.024	0.050
Difficulty × Feedback × Self-Esteem	0.070	1	0.070	2.149	0.148
Residual	1.781	55	0.032		

Note. Type III Sum of Squares

Between Subjects Effects

	Sum of Squares	df	Mean Square	F	p
Self-Esteem	3.297×10^{-6}	1	3.297×10^{-6}	0.003	0.955
Residual	0.056	55	0.001		

Note. Type III Sum of Squares

Table S5. A 2 × 2 × 2 repeated measures ANOVA on end-of-block task choices with Difficulty (Easy, Difficult) and Feedback (Feedback, No Feedback) as within-subject factors and Self-Esteem (high, low) as a between-subject factor. This is the same analysis as reported in Table S2 but with recruitment self-esteem level instead of current self-esteem level.

Within Subjects Effects

	Sum of Squares	df	Mean Square	F	p
Difficulty	0.252	1	0.252	212.335	< .001
Difficulty × Self-Esteem	0.002	1	0.002	1.530	0.221
Residual	0.065	55	0.001		
Feedback	0.326	1	0.326	139.312	< .001
Feedback × Self-Esteem	5.209×10^{-4}	1	5.209×10^{-4}	0.223	0.639
Residual	0.129	55	0.002		
Difficulty × Feedback	0.036	1	0.036	36.428	< .001
Difficulty × Feedback × Self-Esteem	9.113×10^{-4}	1	9.113×10^{-4}	0.912	0.344
Residual	0.055	55	9.993×10^{-4}		

Note. Type III Sum of Squares

Between Subjects Effects

	Sum of Squares	df	Mean Square	F	p
Self-Esteem	0.137	1	0.137	5.046	0.029
Residual	1.492	55	0.027		

Note. Type III Sum of Squares

Table S6. A 2 × 2 × 2 repeated measures ANOVA on end-of-block task ratings with Difficulty (Easy, Difficult) and Feedback (Feedback, No Feedback) as within-subject factors and Self-Esteem (high, low) as a between-subject factor. This is the same analysis as reported in Table S3 but with recruitment self-esteem level instead of current self-esteem level.
